# Medical Microscopy

**Published:** 1903-03

**Authors:** 


MEDICAL MICROSCOPY. Designed for Students in Laboratory Work and for Practitioners. By T. E. OERTEL, M. D., Professor of Histology, Pathology, Bacteriology, and Clinical Microscopy, Medical Department, University of Georgia. Duodecimo, pp. 374. With 131 illustrations, some of which are colored. Philadelphia: P. Blakiston's Son & Co. 1902. [Price, $2.00 net.]

Dr. Oertel presents a work intended both as a laboratory manual and as a guide to the practitioner who is unable to avail himself of a post-graduate course in clinical microscopy. He includes in the volume the subject of bacteriology so far as it pertains to clinical medicine, the examination of the blood, the preparation of sections, and the examination of the same with special reference to malignant growths, the examination of the urine, sputum, and the like. As a laboratory guide the book is an excellent one. It will not teach the solitary student how to become an expert microscopist. It will, however, be as useful as a book can be in teaching him the technic of microscopy. With the beginner ever in mind, Dr. Oertel has presented his subject in the simplest possible manner, giving in most instances but one method of doing a certain thing, and that the one most simple, or least liable to error.

The illustrations are numerous and good.

